# Acute Alveolar Abscess, Its Pathology and Treatment

**Published:** 1913-02-15

**Authors:** Rafael Tarregrosa

**Affiliations:** Porto Rico


### ACUTE ALVEOLAR ABSCESS, ITS PATHOLOGY AND TREATMENT.

BY RAFAEL TARREGROSA, D.D.S., PORTO RICO.

My idea in writing on this important subject is to make a few brief and practical observations, stopping only to discuss some of the points which seem to me are of most importance.

In my opinion, of all the cases that come to a dentist, an acute alveolar abscess is the one requiring the most careful attention, and the most accurate knowledge of the histology and pathology of the parts involved. An abscess of this kind always begins with an inflammation which has a septic origin in the pericemental tissues. The cause of such pericementitis usually has its septic origin from a putrid or infected pulp. The pyogenic germs of an infected pulp produce gases and toxic products, and in the supurative stage, a constant pus supply, all of which become active causes of inflammation in the pericemental tissues of the apical region. We may have infection of the apical tissues by canal instruments, which have not been properly disinfected, or which are carelessly manipulated. Occasionally we have infection from adjoining diseased teeth, or from a pericemental abscess on the affected tooth, or in the near vicinity of the tooth, and extending to the apex by tissue contact. We may have infection from the gums and peridental tissues of pyorrhea teeth, extending also to the apex of the root. This is particularly encountered in pyorrhea conditions, which are very common in Porto Rico, and is one of the chief causes of my troubles. We may also have infection of the peridentium of the tooth by means of the circulation, or by germs reaching the foci through the gums and the pericementum, as suggested by Kirk and Black.

Microscopical investigation of the pus of these abscesses shows that they are not caused by a specific germ, but by several kinds of micro-organisms. Among these, that which we most frequently find, is the diplococcus pneumonia, many times associated with staphylococcus albus and aureus. The streptococcus, which is one of the most harmful micro-organisms, is often the chief factor in the more serious infections. The pathology of these cases involves various periods of morbific changes of the tissues invaded. The first pathological state is caused by a pyogenic infection, causing an inflammation of the tissues around the tooth. This inflammation begins with hyperemia and congestion of the arterioles. The tooth aches and becomes sore and sensitive to temperature changes, and to percussion. All the tissues involved ultimately degenerate and break down into pus. Not only does the connective tissue and mucous membrane degenerate, but the alveolar process becomes infected, and undergoes liquifaction to some extent. As this pus continues to be formed in considerable quantities, the spongy bone of the process around the tooth also degenerates, and the pus will travel along the tissues that offer it the least resistance, to an outlet. The facial tissues in the neighborhood of the tooth swell, and become hardened, but these soon soften, when pus forms, and the pain, if present, will disappear. In the simple cases, the pus escapes from the sac enclosing it by a fistulous canal or sinus, and finds its way to the surface of the gum, or sometimes to the surface of the face. In all cases of alveolar abscesses, there is the danger of absorption of the toxins and products of degeneration into the system by means of the circulation, the symptoms being fever, with chills, increase in pulsation, constipation of the bowels, insomnia, and general debility.

The clinical history is divided into three parts. First, is inflammation, followed by the formation of pus. Second, there is destruction of the alveolar process. Third, the escape of the pus into the spongy bone and overlying gum tissues. In this stage there is a period of induration or oedema. In the majority of the cases, associated with the lower molars, or with the lingual roots of the upper molars, the density and thickness of the bone resists the pressure of the pus, and it is compelled to break through the gum tissue and mucous membrane. When the connective tissues of the gum offers too much resistance, the pus attacks the bone, and necrosis is set up. This is done by dissolution of the periostium, depriving the bone of its nutrition. Preventive treatment in such cases has been the application of emollient ointments, which by softening the tissues, allow the pus to come out through the cheeks, and the neck, leaving a permanent scar. The proper treatment should be applied, immediately after the inflammation and soreness start, for these are definite and sure symptoms. The surgical treatment requires that the root canals be opened to the apex and communication thus established with the pus cavity and the external sinus, if there be one. Then disinfection of the canals as well as the pus pockets and sinus is made possible. An antiseptic agent, like campho-phenol, or some of the essential oils is applied to maintain asepsis. The gums may be painted with a lotion of equal parts of iodin and aconite, to allay superficial irritation. Treatment of this kind will control the inflammation and suppurative process in a couple of days. When the abscess has spread to any considerable extent, and the treatment outlined can not be applied, we are sometimes compelled to lance the gums freely, and if necessary, trephine into the bone to establish an artificial fistula or sinus for drainage. If the abscess has reached its maximum state, the incision must be deep, and the bone and root must be curetted or excised to remove the necrotic conditions. The operated field must be thoroughly sterilized with non-cauterant antiseptic irrigants, such as boric acid, or diluted phenol solutions used warm. We must also establish continuous drainage so that the fistula will begin to heal from the bottom. In more serious cases, such as the one that I had to deal with and which did not get better by these methods, the tooth has to be extracted. Some authorities, such as Black and Kirk, justify such radical treatment, even in the most acute suppurative conditions, although many believe that a general septicemia or pneumonia may ensue from extraction at this period. Alveolar abscesses are often found associated with the temporary teeth. The symptoms and the treatment will be much the same as with adults, although more complicated, for at this age the alveolar process and teeth are still developing.

In infections caused by staphylococci and diplococci or a mixed infection, the prognosis is in the majority of cases favorable. The course of the infection is usually short, and the only complications liable to occur are the infection of the bone, and the antral cavity of the upper jaw. A streptococcus infection is the most serious and troublesome to handle. My opinion is that a dentist with a thorough knowledge of the anatomy of the tissues of the face, who is competent to differentiate the pathology of the case, is much more capable of successfully handling one of these cases than a physician. It is very often that the dentist instead of treating these cases, will send the patient to a physician, who is less apt to know the proper treatment, than the dentist.

When the patient presents a serious hyperemia with much swelling, and great pain about the tooth, first get the history of the case, including all the previous symptoms. The diagnosis can be made on the following outline. First, determine the progress of the inflammation for two days after it became irritable. Second, note its extension to the face, and other associated organs and tissues. Also note carefully if there are referred pains, and to what parts. Third, note the hyperemic coloration of all the inflamed parts. Fourth, note the duration or prolongation of the inflammation with any tendency to invade the associated lymphatics. Observe the general systemic disturbances, especially an abnormal temperature of from 38 to 40 degrees C., due to the slow absorption of the toxins. Sixth, observe any difficulty in localizing the point of infection. The treatment of this form of infection is strictly surgical, and the patient must be prepared for an operation. The first step is to thoroughly disinfect the oral cavity, and contigous parts. The teeth should be thoroughly brushed with an abrasive and disinfectant powder, and the mouth irrigated with a syringe or spray, using a solution of $H2O2$ and afterwards some milder antiseptic solution, and the gum should be bathed with a solution of iodine where the cut is to be made. If a local anesthetic is to be used, great care should be exercised not to penetrate the pus area. This especially, if cocaine is used. An obtundent, such as ethyl chloride spray may best serve the purpose. In some severe cases a general anesthetic must be used. The incision should be made in the place where the pus is suspected of lying. This incision must be deep and of such extent as to secure drainage with the least injury to the nutritional vessels. The pus in these abscesses is dark and mixed with serum and blood and its odor generally extremely offensive. The cavity should be drained and irrigated and then a careful examination of the bone should be made with a probe. In case there is any necrosed bone, it must be curetted or removed, and the wound packed or prepared for subsequent drainage. After the operation, the patient should be advised to rinse his mouth at intervals with an antiseptic solution for several hours. The patient must be looked after every day, until healing is assured, for fear of a secondary infection.

I will illustrate my subject by reciting a serious case, which recently came into my practice. A girl seven years old, with a fair constitution and good health, began to feel a slight pain in the first, temporary lower molar of the left side. The pain increased considerably during the next two days. The third day the pain had ceased, but inflammatory swelling had begun. Her parents did not pay much attention to the case, but they had her wash her mouth with an antiseptic solution. The fourth and fifth day, the inflammation had spread all over the inferior maxillary and a large portion of the face. A physician was called, and he made an incision on the lingual surface, but not deep enough and soon afterward it healed up again. The sixth day I was called by the physician to see the case, and to give my diagnosis. The inflammation had invaded not only the left side of the face, but also a greater part of the sub-lingual region, the neck, and part of the right side. The temperature ranged from 39 to 40 degrees C. The case clearly indicated a streptococcus infection, caused by the molar tooth with the principal focus of infection lying at the apex of the root. This I told the physician, advising him to make a deep cut throughout the whole lingual surface of the inferior maxilla, from the sympthasis to the ramus, and also the extraction of the first and second deciduous molars. Ordered by the physician, I made the incision myself. I found the focus and the discharge of pus was great. I examined the bone carefully, and ordered the mouth washed with glyco-thymoline. The result was very satisfactory, the inflammation diminished, the fever disappeared, and for a period of four days, everything went alright. But the fifth day, another inflammation started in the external angle of the jaw, extending to the parotid region, and endangering the gland. I was called again for the girl was worse than ever, the fever just as high. I examined the primary focus of infection and found it with no pus, and the wound almost healed up. The girl complained of pain in the ear, (without doubt, a reflex action.) We apparently had a secondary abscess, and what seemed to be a new focus of infection. I knew then that the bone must be necrotic. I urged the physician to operate immediately, cutting on the external surface of the angle of the ramus, but we tried to reach the focus from the internal surface first, and so avoid a scar which would be permanent. I made an incision near the anterior pillar of the pharynx, but without result, as I expected. We finally decided to operate externally, and that night we prepared the girl for the anesthetic. At seven o-clock in the morning we began to operate. Before making the incision, we punctured at the region of the ramus, and with a suction syringe drew out a quantity of pus. Then we made the cut deep enough to reach the deposit of pus. The pus was of a dark red color and very fetid. We found the bone necrotic and curetted it thoroughly. We disinfected the wound with a glyco-thymolin wash, and a lotion of tincture of iodin. Then we inserted a rubber tube, covered with gauze, for drainage. After fifteen days the wound was completely healed.

---

